# The National Medical Union (Official)

**Published:** 1914-05-30

**Authors:** 


### THE NATIONAL MEDICAL UNION (Official).

Offices : 346 Strand. Tel. : City 8444.

### FINAL MEETING OF PROVISIONAL FEDERATING COUNCIL.

The final meeting of the Provisional Federating Council was held on Saturday, May 23, at the offices of the Union. Present: Dr. Greenyer in the chair, Drs. Bousfield, Burgess, Greene, Johnston, Spurgin, Wilson, Carswell, and Mr. Dorrell.

Dr. Burgess reported that since the last meeting of Council the Society of Apothecaries had taken action in the case of two herbalists, with the result that in the one instance a fine of £20 had been imposed, and in the other the case which had been postponed would probably result in a similar conviction and fine.. Dr. Burgess also stated with reference to Regulation 44 (2) of the National Insurance Commissioners that the Apothecaries' Society was prepared to consider for prosecution any case in which unqualified attendance was paid for by an Insurance Committee. In any such case the evidence required by the Apothecaries' Society would be an account rendered by a herbalist or bone-setter and paid by the committee.

The report of the Constitution Committee was read by the Hon. Secretaries. In this report it was recommended that the National Medical Union should apply for registration under the Companies Act, 1867, and that the liability of members should be limited by guarantee to £1; it was also recommended that for the membership of the permanent Council the local associations should conduct elections on the basis of one member of Council for every association of twenty-five subscribers and under, with an additional member for every twenty additional subscribers, the minimum membership entitling any society to elect a member being fixed at ten. Associations having under ten members would elect their representatives in common with the unattached members of the Union through the central office. The report was adopted by the Council, and, with the report of the Executive Committee, will be laid before the general meeting, together with other matters.

The date of the general meeting was fixed for Saturday, June 27, the meeting to begin at 10.30 A.M.

A committee consisting of the Chairman of Council (Dr. Greenyer), Drs. Spurgin, Johnston, and Greene, together with the Hon Secretaries, was appointed to draw up the report of the Provisional Council for presentation to the general meeting, to prepare the agenda, and to prepare a prospectus of the Union for general distribution.

A Dinner Committee was also appointed, consisting of Drs. Bousfield, Wilson, Sharman, Hemmans, Johnston, and Edwin Smith.

At the meeting of Council on March 21 a resolution from Wandsworth, to the effect that recognition should be given to the principles—(*a*) that payment of premiums should be made collectively to associations of doctors, and (*b*) that the sum total should be divided by such associations on the basis of payment for work done, was relegated to a sub-committee for consideration and report. A Manchester resolution concerning the system of the National Deposit Friendly Society and a resolution from Edinburgh concerning the Scottish Clerks' Association were also included in the reference. The meeting, however, terminated without appointing the members of the committee. At the Executive Committee meeting on May 2 the subject was discussed informally, and at the Council meeting on Saturday last it was decided to appoint Drs. Greenyer, Brassey Brierley, Oppenheimer, Porter, Greene, Johnston, Sharman, Carswell, and Mr. Dorrell, who had been present at the Executive Committee meeting on May 2, to reconsider the subject as a sub-committee of Council, and report at some date after the general meeting to the permanent Council on the subject. Dr. Greene withdrew his nomination from the committee on the ground that it would be a waste of time to discuss this subject at present, and Dr. Spurgin was appointed in his stead.

Dr. Greenyer expressed the opinion that the whole of these questions would never come forward, and that the policy of the National Medical Union was to fight as hard as possible to reduce the income limit, leaving a State medical service for the residue below the reduced limit. The policy of the Union should be to have nothing to do with insurance companies. Consideration of these resolutions was therefore premature.

Mr. Dorrell pointed out that notwithstanding such reduction of income limit there would still be a number of people within the possibility of coming under the old conditions of contract medical attendance. The societies would endeavour to make business and capital out of the old conditions which the medical profession might have to be prepared to meet. In this case the medical profession could not return to the conditions of service of the past. The difference between what had formerly existed and what was embodied in these resolutions was the difference between individual and collective bargaining.

The Council, in appointing the above committee, instructed it to report to the permanent Council when elected at some date subsequent to the general meeting.

### Dinner Committee.

A meeting of the Dinner Committee took place at the offices of the Union on Tuesday, May 26, when it was decided that the dinner should be held at the Café Monico. Piccadilly Circus. It is expected that there will be a large attendance of members of the Union, but non-members who are in sympathy with the objects and policy of the Union are to be invited. A further notice on the subject will be found in next week's issue of The Hospital.

### THE PROFESSION OF MEDICINE.

### A Short Way with a Venal Press.

It is an old story that lay newspapers—not all, but most of them—cannot be induced to give publicity to the misdeeds of fraudulent quacks because of the large sums spent annually by the latter on advertisements. With a few honourable exceptions, for instance, the press boycotted the British Medical Association's two valuable books on "Secret Remedies"; and would neither review them nor insert advertisements regarding them. In New York the almighty dollar leads to a similar state of affairs; and, again, as in Britain, the sexual quack is he whose purse is the deepest and his pretensions the most extravagant. The Medical Adviser to the Department of Health, Dr. B. S. Barringer, has been taking a bold line with these gentry; and what he has done might well be imitated over here. Three months ago he read a paper to a local medical society, and he has now published it in the *Long Island Medical Journal.** In this paper he gives particulars of the revenue derived annually by leading New York newspapers from quack advertisements dealing with venereal disease, abortion, and so on. Four of them, it appears, derive over ten thousand dollars a year each from this tainted source, the largest sum accruing to any one paper being $19,696. Eight more receive between one and ten thousand dollars apiece; and several others take lesser sums. This list, too, represents only one half, or possibly a little more, of the advertisements of this type which appear in New York newspapers according to Dr. Barringer.

But publicity alone will not check the abuse. Something more is required, as experience in this country shows. The Medical Adviser has accordingly carried the war into the enemies' country, a proceeding which has aroused certain misgivings among his medical colleagues in private practice. He has, however, little difficulty in proving that he is doing good. He picked out that daily paper (the *Evening Telegram*, to be exact) which had the most quack advertising, and he paid for an advertisement offering free advice regarding venereal disease at the Departmental Offices. He found that of those who came for advice about 90 per cent. had previously consulted advertising quacks without avail; some had been to as many as fifteen unqualified practitioners. He came across numerous cases of gross *malpraxis*, naturally, and a good many of incorrect diagnosis. The illustrative cases which he quotes are exceedingly interesting specimens of the methods of the sexual quack. One of their favourite devices is to charge high fees for the injection of coloured water which is alleged to be salvarsan solution: indeed, some of them do not even inject anything, but allow the coloured fluid to escape unseen into a bucket.

Dr. Barringer then discusses ways and means of putting a stop to these quack advertisements. In Oregon, he notes, an ordinance has been put in force severely penalising both the advertisers and those who accept their advertisements. The wording of the ordinance runs: "Any person who shall advertise or publish any advertisement intended to imply or to be understood that he will restore manly vigour, treat or cure lost manhood, lost power, stricture, gonorrhœa, chronic discharges, gleet, varicocele, or syphilis. . . ." There seems to be some slight doubt about the constitutional legality of this ordinance; but this point has never yet been tested in the Courts, owing to the fear of publicity, to which anyone who objected to it would certainly attain. Further, we have Dr. Barringer's word for it that the ordinance has eliminated the class of advertisement against which it was directed. In Chicago, mainly as a result of a campaign carried on by the *Chicago Tribune*, an ordinance has been passed which has much the same effect, though it is quite differently worded. And in New York, the Medical Adviser and others are hoping to get a similar ordinance established. There is ample room for some similar restrictions on quackery in Great Britain, though probably national rather than local legislation would suit the conditions best. Nor are venereal diseases alone favoured by the unqualified advertiser. Deafness is another of his stand-bys.

* April 1914, p. 144.

### The Re-education of the Deaf.

Some years ago wonderful accounts used to emanate from Paris and Vienna about marvellous mechanical contrivances which would restore hearing to the totally deaf, whether their deafness was congenital or the result of disease. Ear specialists in this country proved somewhat sceptical, as indeed they were in duty bound to be; and the result is that unqualified persons flood the land with pamphlets containing the most extravagant promises, and fleece the public unmercifully. In the *Lancet* Mr. Richard Lake summarises his experience with these methods of so-called re-education. In cases of acquired deafness he confesses to an "appallingly small amount of success." He has come to the conclusion that in cases of acquired deafness this method of treatment rarely offers any hope for those patients whose deafness does not yield to properly and judiciously applied remedies. On the other hand, it is pleasing, he states, to be able to say that at any rate one variety of one re-educational instrument is of benefit in stimulating the dormant power of hearing in some of the congenitally deaf. Educational treatment of those born deaf is by no means a new idea; and one very probable explanation of the fact that it sometimes has a fair amount of success in childhood is that in certain cases there is a delayed development of the auditory centre in the brain. As this centre develops, as it occasionally does in late childhood, a rational educational process may be of the very greatest value in tuning it to a higher pitch.

